# Occupational Segregation And Hypertension Inequity: The Implication Of The Inverse Hazard Law Among Healthcare Workers

**DOI:** 10.1007/s41996-022-00098-5

**Published:** 2022-03-22

**Authors:** Tongtan Chantarat, Eva A. Enns, Rachel R. Hardeman, Patricia M. McGovern, Samuel L. Myers, Janette Dill

**Affiliations:** 1 Division of Health Policy and Management, School of Public Health, University of Minnesota, Minneapolis, MN 55455 USA; 2 Center for Antiracism Research for Health Equity, School of Public Health, University of Minnesota, Minneapolis, MN 55455 USA; 3 Division of Environmental Health Sciences, School of Public Health, University of Minnesota, Minneapolis, MN 55455 USA; 4 Hubert H. Humphrey School of Public Affairs, University of Minnesota, Minneapolis, MN 55455 USA

**Keywords:** Occupational segregation, Hypertension, Healthcare workers, Microsimulation

## Abstract

In the United States (US), Black—particularly Black female—healthcare workers are more likely to hold occupations with high job demand, low job control with limited support from supervisors or coworkers and are more vulnerable to job loss than their white counterparts. These work-related factors increase the risk of hypertension. This study examines the extent to which occupational segregation explains the persistent racial inequity in hypertension in the healthcare workforce and the potential health impact of workforce desegregation policies. We simulated a US healthcare workforce with four occupational classes: health diagnosing professionals (i.e., highest status), health treating professionals, healthcare technicians, and healthcare aides (i.e., lowest status). We simulated occupational segregation by allocating 25-year-old workers to occupational classes with the race- and gender-specific probabilities estimated from the American Community Survey data. Our model used occupational class attributes and workers’ health behaviors to predict hypertension over a 40-year career. We tracked the hypertension prevalence and the Black–white prevalence gap among the simulated workers under the staus quo condition (occupational segregation) and the experimental conditions in which occupational segregation was eliminated. We found that the Black–white hypertension prevalence gap became approximately one percentage point smaller in the experimental than in the status quo conditions. These findings suggest that policies designed to desegregate the healthcare workforce may reduce racial health inequities in this population. Our microsimulation may be used in future research to compare various desegregation policies as they may affect workers’ health differently.

**Supplementary Information:**

The online version contains supplementary material available at 10.1007/s41996-022-00098-5.

## Introduction

Multidisciplinary scholars have observed health inequities between Black and white workers for as long as the data have been collected. However, during the COVID-19 pandemic, the evidence of these inequities has been brought to the spotlight (Chen et al., 2021; Hawkins, 2020; Matthay et al., 2021; Reitsma et al., 2021). Recent data show that Black workers are more likely than their white counterparts to work in essential industries (e.g., healthcare, social assistance, or meat processing) and perform tasks in an environment with frequent exposure to infections with limited protective equipment (Hawkins, 2020). Racialized exposures to occupational health risks and workers’ health inequities are not new phenomena nor specific to COVID-19. To eliminate workers’ health inequities, researchers and policymakers must critically examine the role of work as a social determinant of health (Ahonen et al., 2018; Lipscomb et al., 2006).

The inverse hazard law characterizes that exposure to occupational health risks is inversely linked to workers’ power (Krieger et al., 2008), which can be proxied by workers’ occupational status in the organizational hierarchy. High-status workers—those with high education and high skills—receive high wages and can muster resources to avoid exposure to occupational hazards. They also face a lower risk of job loss and are less likely to lose access to health-promoting resources than low-status workers. In addition, although high-status workers tend to have a higher job demand, their job tends to offer higher levels of control over what, when, and how they conduct their tasks, which further minimizes work-related stress that could have negatively affected their health (Gilbert-Ouimet et al., 2014; Marmot et al., 1991). Because work influences health status through access to social and material resources and patterns of exposure to high-risk work conditions, occupational segregation is a fundamental driver of racial health inequities (National Academies of Science, Engineering, and Medicine, 2017). However, interventions to eliminate workers’ health inequities have focused primarily on redesigning jobs or changing workplace environments to minimize occupational health risks, with limited attention given to structural changes to eliminate occupational segregation (Ahonen et al., 2018; Lipscomb et al., 2006).

This study uses a microsimulation to examine the extent to which the elimination of occupational segregation in the workforce narrows the racial gap in workers’ health. We used hypertension among healthcare workers as a case study. This paper is organized as follows. In the background section, we start by describing the concept of work as a social determinant of health and reviewing broadly the mechanistic linkages between work and hypertension in the current literature. Next, we discuss the inverse hazard law, relating hypertension gradient to predicted patterns of work-related exposure among healthcare workers by occupational classes. Subsequently, we discuss our microsimulation experiment in the method section. Next, we describe hypertension prevalence trends under four hypothetical desegregation scenarios, comparing those to the trend from the occupational segregation simulation. Finally, we discuss the real-world impact and potential implications for future policies.

## Background

### Work as a Social Determinant of Health

Work is a determinant of health and a driver of population health inequities (Ahonen et al., 2018). A multi-faceted concept, “work” encompasses individuals’ ability to get work (employment status), the type of work (occupation), the setting of work (industry), the nature of work (e.g., physicality, duration of a shift), and the workplace environment (e.g., exposure to environmental hazards and the psychosocial work environment). Because of this complexity, the mechanism underlying the social production of workers’ health is often studied separately by organizational sociologists, labor economists, and occupational health scholars from varying disciplinary perspectives. With limited integration of these perspectives, evaluating the health effects of work from policies that address distal causes (e.g., what happens during the labor force development) is challenging and a critical gap in the literature.

In this study, we used hypertension as an example of a highly prevalent chronic health condition among the US workforce to examine the extent to which occupational segregation and racialized exposure to occupational health risks contribute to hypertension inequity between Black and white workforces. Combing all occupations in the US economy, hypertension affects 27% of Black workers and 21% of white workers (National Institute for Occupational Safety and Health, 2019). Hypertensive workers face an increased risk of cardiovascular diseases, stroke, and preventable death (Benjamin et al., 2018). On average, hypertensive workers incur 2.5 times higher in-patient care expenditures and spend three times as much on prescriptions than workers with normal blood pressure (normotensive) (Kirkland et al., 2018). Hypertensive workers also report more absenteeism than their healthy peers, which leads to lost productivity and affects their coworkers in team-based workplaces (Unmuessig et al., 2016).

Past research has shown several ways that work influences the risk of hypertension. Work acts as a “gatekeeper” to resources needed for workers’ health and well-being (e.g., wage and material resources, healthcare coverage, leisure time) (Lipscomb et al., 2006). The role of work as a gatekeeper to health-promoting resources is especially evident during economic recessions. For example, during the 2007 to 2009 recession, the US unemployment rates rose from 5% to 9.5%, and the median household income dropped from $54,489 to $52,195. Approximately 3 million Americans lost employer-sponsored healthcare coverage, and there was a significant decline in US population health metrics (Bureau of Labor Statistics, 2012; Burgard & Kalousova, 2015; Holahan, 2011; Kochhar, 2012). In addition, unemployed workers were less likely to maintain a healthy diet, not meet recommended guidelines for physical activities, and were more likely to smoke and consume alcohol heavily during the recession (Compton et al., 2014; Kwak et al., 2016; Smed et al., 2017; Van Domelen et al., 2011), further reiterating the fact that the loss of work leads to an increased risk of hypertension.

Besides being a resource gatekeeper, work also acts as a stressor that affects blood pressure directly via the stress pathway. Workers who have experienced job loss and those whose jobs are unstable or precarious are more likely to report hypertension than those with stable jobs (Benach et al., 2014; Kaur et al., 2014). Older male workers whose employer reorganized or downsized and those who reported being disciplined or demoted—an indicator of job insecurity—reported systolic blood pressure (SBP) 5.2 mmHg higher than their counterparts whose job was secure (Kalil et al., 2010). A slightly lower but significant increase in SBP was reported among female workers experiencing job insecurity (Ferrie et al., 1998). Studies from the US and many European countries also reported higher SBP and diastolic blood pressure (DBP) among workers experiencing job loss than employed workers. The detrimental effect of job loss on hypertension risk varies based on the workers’ gender, income level, age when job loss occurs, and the duration of the unemployment (Acevedo et al., 2019; Leigh and Du, 2012; Levenstein et al., 2001; Nygren et al., 2015).

However, not all work is created equal. The benefits of work on cardiovascular health are modified by the work-related stress that arises from poor working conditions and the workplace environment. Among the many dimensions of work that affect workers’ hypertension risk, researchers have consistently demonstrated the linkage between work-related psychosocial stressors (“psychosocial work environment”: PWE) and hypertension status (Gilbert-Ouimet et al., 2014). PWE is characterized by job demands (workload, time pressure, and role conflict), job control (decision authority, skill discretion, and the worker’s ability to control their work activities), and support from coworkers and superiors (Johnson & Hall, 1988; Karasek, 1979). According to the Job Demand-Control-Support model (Johnson & Hall, 1988; Karasek, 1979), while workers with high job demand are more likely to experience stress/burnout, job control can promote learning and mitigate job demand’s harmful effects. Workers with high-demand, low-control jobs (i.e., job strain) have an increased risk of stress-related, adverse health conditions, while those with low-demand, high-control jobs have the lowest risk. For a given level of job strain, the level of superior and coworker support further protects against the increased risk of hypertension; workers holding jobs with high demand, low control, and low support (i.e., iso-strain) face the greatest risk of hypertension (Johnson & Hall, 1988; Karasek, 1979).

### Power, Occupational Class, and Hypertension Inequity

Work-related risks of hypertension are patterned by workers’ occupations (Landsbergis et al., 2015). The inverse hazard law posits that “the accumulation of health hazards tends to vary inversely with the power and resources of the populations affected” (Krieger et al., 2008). Consistent with this theory, workers in high-status occupations (i.e., having high education, high skills, and high earning) can muster materials and social resources to avoid exposure to health risks as well as create or influence institutional rules, policies, and practices to sustain their dominant position in the organizational hierarchy (Budig et al., 2019; Krieger et al., 2008; Robinson, 2000). Early evidence of health gradients among workers by occupational class originated from the Whitehall study (Marmot et al., 1991). Among British civil servants with the same access to government-sponsored healthcare, workers in lower employment grades were more likely to be hypertensive and had a higher mortality risk of coronary heart disease (Marmot et al., 1991). A similar trend was also observed among US workers in many industries. For example, male aluminum manufacturing plant workers in the lowest income tertile is 1.2 times more likely than those in the highest income tertile to have hypertension. For female aluminum workers, the odds of hypertension for those in the middle income tertile is 1.3 times higher than those in the highest income tertile (Clougherty et al., 2011). Among healthcare workers, the hypertension prevalence was 14% in health diagnosis professionals (e.g., doctors, dentists), 24% in health treating professionals (e.g., registered nurses, physicians assistants), 28% in healthcare technicians (e.g., laboratory technicians, dental hygienists), and 36% in healthcare aides (e.g., nursing aides, dental assistants), demonstrating the risk gradient by workers’ occupational class and power within healthcare organizations (Lee et al., 2012).

The observed hypertension gradient is attributable to the pattern of exposure to work-related risks of hypertension by occupational class. Compared to high-status workers, low-status workers face a higher threat of job loss and job insecurity. For example, the unemployment rate for US workers in the “management, business, and financial operations occupations” is 2%. In contrast, the unemployment rates for workers in the “service occupations” and “transportation and material moving occupations” are approximately 5% (Bureau of Labor Statistics, 2020). Increased unemployment rates by occupational class are also observed across workers within the same industry. For example, the unemployment rates among health diagnosis professionals, health treating professionals, health technologists, and healthcare aides are 1%, 1%, 3%, and 4%, respectively (Ruggles et al., 2020). Besides being more vulnerable to losing access to material and social resources brought about by unemployment, low-status workers are more likely to experience iso-strain than high-status workers, the trends dated back to the 1990s in many countries (Aust et al., 2007; Gallo et al., 2004; Joseph et al., 2016; Light et al., 1992; Marmot et al., 1991).

Altogether, this evidence shows how an oppressive system that segregates certain workers into particular occupations or occupational classes and systematically exposes the oppressed group to a disproportionate level of hypertension risks than others is one of the fundamental drivers of hypertension inequity in the workforce (National Academies of Sciences, Engineering, and Medicine, 2017). Occupational segregation is a product of two forms of oppression: structural racism (i.e., racist ideologies, policies, and practices that reinforce one another to inhibit access to economic opportunities for non-white workers), intertwining with structural sexism (i.e., ﻿ “the systematic gender inequality in power and resources manifest in a given gender system”) (Bailey et al., 2017; Chung-Bridges et al., 2008; Gee & Ford, 2011; Homan, 2019).

### Occupational Segregation and Hypertension Inequity in the Healthcare Workforce

**Fig. 1:**
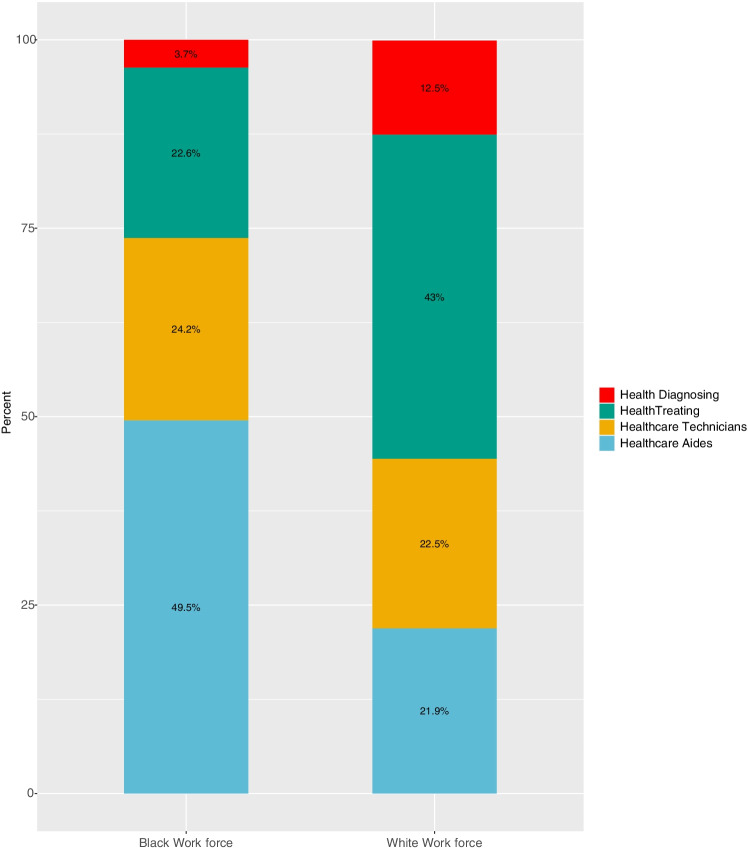
Proportional representation of four occupational classes in the white and Black healthcare workforces

A highly segregated healthcare workforce mirrors racial segregation in other parts of our society, clustering Black workers, particularly Black female workers, disproportionately at the bottom of the occupational hierarchy. **Figure ****1** shows workers’ distribution by occupational class for the Black and white healthcare workforces in the US in 2017 based on the American Community Survey (ACS) data. We grouped healthcare occupations using the Standard Occupational Code (SOC) into health diagnosing professionals, health treating professionals, healthcare technicians, and healthcare aides based on the level of education required to perform healthcare tasks. For specifics SOCs in each group, refer to the **Online Resource 1**. Compared to non-healthcare occupations, credentialing systems highly regulate healthcare tasks (Duffy, 2011; Wingfield, 2019). Healthcare workers must obtain the necessary formal education and professional training and pass licensing tests to enter into specific professions. Professionalization of healthcare tasks prevents workers from climbing the occupational ladder as they “learn on the job,” restricting interclass mobility and sustaining occupational segregation in the healthcare workforce, particularly for workers of color (Ray, 2019).

Occupational segregation in healthcare organizations likely contributes to racial health inequity in the healthcare workforce. This less discussed linkage became increasingly evidenced during the COVID-19 pandemic. Healthcare workers in close contact with infectious patients are more likely to have low wages, limited access to paid time-off, and little control of their work activities; they are also more likely to be Black (Hawkins, 2020). For hypertension, our analysis of the National Health Interview Survey (2014–2018) shows the risk gradient was also inversely related to the occupational hierarchy. For Black healthcare workers, the hypertension prevalence is 34%, 34%, 40%, and 40% for health diagnosing professionals, health treating professionals, healthcare technicians, and healthcare aides. A similar trend was observed among white healthcare workers (28% for health diagnosing professionals, 12% for health treating professionals, 30% for healthcare technicians, and 30% for healthcare aides); however, the hypertension prevalence of all occupational classes is significantly lower compared to Black healthcare workers. Further details of this analysis are available in the **Online Resource 2**.

### Current Study

Integrating information from organizational sociology, labor economics, and occupational health sciences literature, we used a microsimulation model to examine the extent to which occupational segregation contributes to racial inequity in hypertension in the healthcare workforce. Microsimulations integrate mathematical equations that describe biological and social processes with factors that determine behaviors and health outcomes under various conditions. This analytical approach does not require participant recruitment. Instead, a computer program simulates a cohort of people with similar characteristics as the population of interest and tracks changes in socioeconomic, behavioral, and health trajectories over their life course. Researchers can program simulated people to adapt their behaviors and make health-related decisions rationally based on their experience (Kaplan, 2017; Luke & Stamatakis, 2012; Macy & Willer, 2002; Marshall & Galea, 2015; Tracy et al., 2018). Compared with “gold-standard” causal inference methods like randomized controlled trials (RCTs), where one group of participants receive standard treatment and the other group with similar characteristics receives experimental treatment, the whole simulated cohort can be in both controlled and experimental conditions. Because of this flexibility, microsimulations can control for the effects of unmeasured variables as well, or arguably better than RCTs (Marshall & Galea, 2015).

For this particular study, we created a microsimulation that mimics the dynamics of employment status and PWE along with health behaviors to predict hypertension pathogenesis for Black and white workers across four healthcare occupational classes. Using a counterfactual scenario analysis, we examined the hypertension prevalence trends among Black and white healthcare workforces to determine the extent to which desegregation of the healthcare workforces decreases the Black–white inequity in hypertension prevalence. Results from our experiment inform policymakers of potential implications on racial health inequities among healthcare workers resulting from implementing effective workforce desegregation policies (Kalev et al., 2006).

## Materials and Methods

### Microsimulation

We simulated healthcare workers’ hypertension development and progression using a state-transition model (STM). STMs conceptualize dynamic processes (e.g., disease development) as a series of conditions (“states”) and events (“transitions”) that individuals experience over time. In each time step (“cycle”), individuals can move from one state to another based on some likelihood (“transition probabilities”), which can depend on an individual’s characteristics and past events. These transition probabilities are from published clinical or observational studies. Researchers can also estimate them from representative population data. After the population of simulated individuals transitions for the desired number of cycles (“time horizon”), the number of simulated individuals in each state at each cycle is tallied to calculate disease prevalence and other outcomes of interest over time (Siebert et al., 2012).

**Fig. 2:**
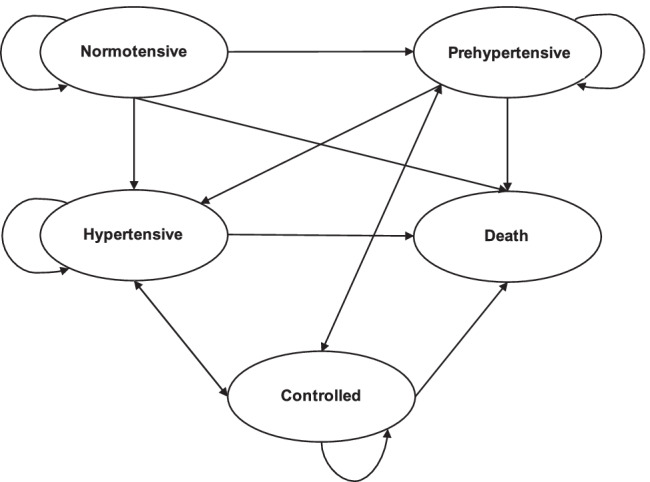
Hypertension state transition diagram

**Figure ****2** shows the dynamic process of hypertension development and progression used for our model. We tracked the hypertension status of simulated workers from age 25 (when average US workers have attained their terminal educational degree) to the pre-retirement age of 64. In our model, one cycle represents one year. Over the workers’ 40-year career, they may be in one of the five health states: *normotensive* (SBP < 120 mmHg and DBP < 80 mmHg), *prehypertensive* (SBP of 120–139 mmHg or DBP of 80–89 mmHg), *hypertensive* (SBP > 140 mmHg, DBP > 90, or being prescribed a hypertension medication), *controlled* (being hypertensive in the previous cycle but normotensive in the current cycle), and *dead*. The blood pressure thresholds used to define these states were based on the Seventh Report of the Joint National Committee on Prevention, Detection, Evaluation, and Treatment of High Blood Pressure (Chobanian et al., 2003).

Over time, normotensive workers can remain normotensive, become prehypertensive, or become hypertensive. Prehypertensive workers can remain prehypertensive, become hypertensive, or become controlled. The transition probability between the prehypertensive and controlled states is assumed to be the same as if workers receive behavioral modification advice (i.e., lose weight, reduce sodium intake, increase physical activity, use the Dietary Approaches to Stop Hypertension (DASH) diet) by their healthcare providers. Prehypertensive workers cannot return to the normotensive state. The probability for prehypertensive workers to become hypertensive is higher than for normotensive workers (Egan & Stevens-Fabry, 2015). Hypertensive workers can remain hypertensive or become controlled. The latter transition is conditioned on the workers’ access to hypertension medication and levels of adherence. Black hypertensive workers receive hypertension medications by healthcare providers at a higher probability than their white counterparts (Samanic et al., 2020). Our model assumes that after hypertensive workers receive hypertension medications, they will remain on those medications as long as they remain hypertensive. For hypertensive workers who are not prescribed medications by their providers, we assume they have the same probability of receiving medication in the following cycle as when they first become hypertensive. Hypertensive workers may adhere to medications at three levels: high (medication adherence rate (MPR) > 0.80), medium (MPR of 0.50–0.79), and low (MPR < 0.50). Each adherence level is associated with the probability of blood pressure control. Hypertensive workers who are not taking medications were assumed to have zero probability of becoming controlled. Workers with controlled blood pressure can remain controlled, become prehypertensive, or become hypertensive similar to normotensive workers. All workers may die during their careers from causes related to or unrelated to cardiovascular diseases (CVD). Compared to normotensive, prehypertensive, and controlled workers, hypertensive workers face a higher risk of CVD-related death but have the same risk of non-CVD death. Regardless of the health states, female workers face an additional risk of death from pregnancy-related causes. **Table **1 shows the data source and specific literature from where the transition probabilities used in our model were estimated or directly drawn.

**Table 1:** Model parameters

Parameter	Sample	Source
Transition probability: Normotensive → Prehypertensive	CARDIA participants Year 15	CARDIA
Transition probability: Normotensive → Hypertensive	CARDIA participants Year 15	CARDIA
Transition probability: Prehypertensive → Hypertensive	CARDIA participants Year 15	CARDIA
Transition probability: Prehypertensive → Controlled		Appel 2003
Transition probability: Hypertensive → Controlled (by adherence level)		Bramley 2006
Probability of being prescribed hypertension medication (by race)		Samanic 2020
Probability of low, medium, high medication adherence among hypertensive individuals		Bramley 2006
Mortality rates for CVD, non-CVD, and pregnancy-related cause (by race and gender)		National Vital Statistics System 2017
Hazard ratio for CVD deaths for hypertensive individuals (by gender)		Franco 2005
Transition probability: Physically inactive ↔ Physically active		Dalziel 2006
Transition probability: Never smoker → Smoker ↔ Quit (by age group: 18–29; 30–44; 45 and older)		Yi 2017
Probability of having a parental history of hypertension (by race)		Muntner 2010
Proportion of women in the healthcare workforces (by race)	Healthcare workers aged 25	ACS 2012–2017
Probability of being a current smoker, former smoker, never smoker (by race and gender)	Individuals aged 24	NHIS 2000–2018
Distribution of body mass index (by race and gender)	Individuals aged 23	NHIS 2000–2018
Probability of being normotensive, prehypertensive, and hypertensive (by race and gender)	Individuals aged 25	NHANES 1999–2018
Probability of being physically active (by race and gender)	Individuals aged 24	NHIS 2000–2018
Probability of working full-time, part-time, and unemployed (by race, gender, and occupational class)	Healthcare workers aged 25	ACS 2012–2017
Distribution of job demand, job control, and support (by occupational class)	Healthcare workers aged 25	ACS 2012–2018 linked with O*Net 3.1

In addition, we estimated the probability that workers transition to the prehypertensive and hypertensive states as a function of individual characteristics and behaviors using risk Eqs. (1) and (2). We estimated these equations with the data from the Coronary Artery Risk Development in Young Adults (CARDIA) study (Friedman et al., 1988). Details about our analytical sample, prediction model estimation, and validation approaches are available in the **Online Resource 3**. We converted the estimated five-year probabilities to one-year probabilities using the Declining Exponential Approximation to Life Expectancy (DEALE) method (Beck et al., 1982).

1 $$Logit(5-year hypertension)=-12.07+2.497LogBMI+0.775Current Smoker+1.710Currently Prehypertensive+0.656Family History of Hypertension-0.059Work Full Time-0.135Work Part Time+0.167Job Demand-0.347Job Control-0.160Support+0.024Age+0.197Women$$

2 $$Logit\left(5-year prehypertension\right)=-5.106+0.013LogBMI-0.424Current Smoker+1.155Currently Prehypertensive+0.509Family History of Hypertension+0.361Work Full Time+1.470Work Part Time-0.278Job Demand-0.657Job Control-0.206Support-0.078Age+0.004Women$$

Our model also tracked changes in exposure to hypertension risk factors in Eqs. (1) and (2) in every cycle to determine hypertension status for the simulated workers. As the simulation progresses, the workers become one year older at the end of each cycle. Besides age, smoking status, physical activity, body mass index (BMI), and employment status may change from one cycle to the next throughout the simulation period. Details of how we modeled the dynamics of these characteristics are available in the **Online Resource 4**.

### Simulated Cohorts of Black and White Healthcare Workers

We simulated 500 cohorts of 100,000 Black and 100,000 white healthcare workers for our analysis. The results we reported in this paper are unweighted means from all cohorts. We characterized the simulated workers in each cohort by age (25 years old in the first cycle and, if alive, increased one year at the end of each cycle), gender (male; female), family history of hypertension (have at least one parent with hypertension; no family member with hypertension), BMI (log-transformed for use in the risk equations), smoking status (current smoker; non-smoker/quit), prehypertension status, occupational class (health diagnosing professionals; health treating professionals; healthcare technicians; healthcare aides), employment status (work full-time; work part-time; unemployed), and the three dimension of the PWE associated with the workers’ occupational class. We also characterized the workers’ physical activity (physically active; physically inactive) because it is one predictor of their BMI. We assigned the baseline values for these characteristics using random sampling informed by the probabilities of such characteristics either among healthcare workers or a representative sample of individuals in the labor force (**Table **1).

Our model allocates the Black and white healthcare workers to one of the four occupational classes using the predicted probabilities estimated from the ACS data (2012–2017). This process mimics how workers “choose” their occupation class given their qualifications, social identities, and occupational advantages. For a simulation of an occupationally segregated workforce (i.e., a status quo condition), these probabilities were estimated with multinomial regression conditioned on age, gender, and race of the worker (Eqs. (3) to (5)).

3 $$\mathrm{ln}\left(\frac{\mathit{Pr}\left(health diagnosing\right)}{\mathit{Pr}\left(healthcare aides\right)}\right)={\alpha }_{1a}+{\beta }_{1a}Age+{\gamma }_{1a}Women+{\pi }_{3a}Black$$

4 $$\mathrm{ln}\left(\frac{\mathit{Pr}\left(health treating\right)}{\mathit{Pr}\left(healthcare aides\right)}\right)={\alpha }_{2a}+{\beta }_{2a}Age+{\gamma }_{2a}Women+{\pi }_{2a}Black$$

5 $$\mathrm{ln}\left(\frac{\mathit{Pr}(healthcare technicians)}{\mathit{Pr}\left(healthcare aides\right)}\right)={\alpha }_{3a}+{\beta }_{3a}Age+{\gamma }_{3a}Women+{\pi }_{3a}Black$$

We assume that once the workers “choose” their occupational class at age 25, based on the probabilities shown in **Table **2 (status quo scenario), they will remain in that class until retirement. Employment status may change between working full-time, part-time, or being unemployed throughout the workers’ career, depending on their age, gender, race, history of unemployment, and occupational class (for specific employment status equations, see the **Online Resource 4**). Because there is no interclass mobility in our model, PWE levels associated with the workers’ chosen occupation were assigned once at age 25 by randomly sampling from their empirical distributions for the US healthcare workers (see the **Online Resource 5**). During the follow-up period, PWE was used to estimate transition probabilities only when the workers were employed full-time or part-time. When the workers were unemployed, the model set the exposure to PWE for that particular cycle to zero.

**Table 2:** Predicted probabilities of being in each occupational class for 25-year-old healthcare workers under the status quo and four desegregation scenarios

Scenario	Race and Gender	Health Diagnosing	Health Treating	Healthcare Technicians	Healthcare Aides
**Status Quo Scenario**	White men	0.312	0.235	0.291	0.162
	Black men	0.109	0.151	0.33	0.409
	White women	0.055	0.409	0.252	0.283
	Black women	0.015	0.205	0.223	0.557
**Scenario A**^a^	White men	0.274	0.221	0.299	0.206
	Black men	0.274	0.221	0.299	0.206
	White women	0.046	0.363	0.246	0.346
	Black women	0.046	0.363	0.246	0.346
**Scenario B**^b^	White men	0.087	0.337	0.255	0.321
	Black men	0.087	0.337	0.255	0.321
	White women	0.087	0.337	0.255	0.321
	Black women	0.087	0.337	0.255	0.321
**Scenario C**^c^	White men	0.312	0.235	0.291	0.162
	Black men	0.312	0.235	0.291	0.162
	White women	0.055	0.409	0.252	0.283
	Black women	0.055	0.409	0.252	0.283
**Scenario D**^d^	White men	0.312	0.235	0.291	0.162
	Black men	0.312	0.235	0.291	0.162
	White women	0.312	0.235	0.291	0.162
	Black women	0.312	0.235	0.291	0.162

a. Improved access to high-status occupational classes for Black workers, with improved access to low-status occupational classes for white workers; gender inequity still exists

b. Improved access to high-status occupational classes for Black workers, with improved access to low-status occupational classes for white workers; gender inequity no longer exists

c. Black men access occupational classes similarly to white men in the status quo scenario, while Black women access occupational as white women

d. All workers access occupational classes like white men in the status quo scenario.

### Occupational Desegregation and Hypertension Inequity

Simulation outputs from the status quo model and those that emerged from the models in which one particular model component is modified can be treated as “control” and “experimental” conditions, respectively. In this analysis, we calculated hypertension prevalence as the number of hypertensive workers divided by the number of alive workers for all cycles. See **Online Resource 6** for more information about hypertension prevalence calculation for the status quo simulation. We compared the hypertension prevalence trends among Black and white healthcare workers observed in the segregated workforce to the trends that emerged from four desegregation scenarios. These counterfactual scenarios differ from one another by the probabilities we used to allocate the 25-year-old Black and white workers to occupational classes (**Table **2, scenario A-D).

In *scenario A*, we allocated Black and white workers to occupational classes using race-independent population average probabilities. These probabilities were estimated similarly to those from Eqs. (3) to (5), except we omitted race from these regressions. In this scenario, Black workers have greater access to high-status occupation classes, while white workers have greater access to low-status occupational classes relative to the status quo scenario. However, because we did not omit gender from these regressions, men still have a better chance of entering high-status occupational classes than women. In *scenario B*, we omitted both race and gender from Eqs. (3) to (5). The results of this estimation were race-and-gender-independent population average probabilities. Unlike in scenario A where gender inequity still exists, all workers in scenario B, regardless of their race and gender, can enter a given occupational class with the same probabilities.

We also predicted the Black–white hypertension prevalence trends under the scenarios where improving access to high-status occupations classes for Black and female workers do not directly affect white and male workers. In *scenario C*, we set the probabilities with which Black male and Black female workers access the various occupational classes to be the same as white male and white female workers in the status quo scenario, respectively. In *scenario D*, we allocated all workers to occupational classes with the probabilities experienced by white men in the status quo scenario. Since the only difference between the status quo scenario and each counterfactual scenario is how workers access their occupational class earlier in their career, any difference in the hypertension prevalence trends that emerged from the simulations can be attributed directly to occupational desegregation.

### Sensitivity Analyses

Given that the number of data points (e.g., simulated workers) in microsimulation studies is synthetic, calculating standard errors and testing of statistical significance are not appropriate approaches for this type of study (Eddy et al., 2012; Murray et al., 2017). Instead, sensitivity analyses are conducted to examine the simulation outputs emerged under ranges and sampling probabilities for key model parameters. For our study, we conducted one-way sensitivity analyses to incorporate uncertainty around two parameters determining the transition between the hypertensive and the controlled states: 1) the race-specific probabilities of being prescribed hypertension medication, and 2) the probabilities of blood pressure control by levels of medication adherence. We reran the simulations with these parameters at ± 25% of the values drawn from the published literature (separate runs) and calculated the uncertainty intervals. Although uncertainty intervals are not analogous to confidence intervals, they provide some insight into the range of possible outcome distributions consistent with the model variations.

All analyses were conducted in R version 4.0.2. Our model incorporates an R function *samplev*, which was created by the Decision Analysis in R for Technologies in Health (DARTH) workgroup (Jalal et al., 2017).

## Results

**Fig. 3:**
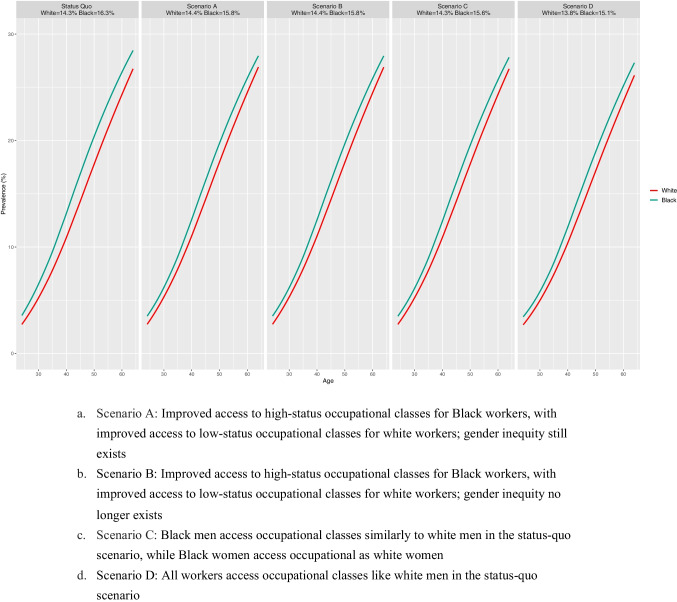
Comparison of the Black–white hypertension prevalence under the status quo and four desegregation scenarios. Scenario A: Improved access to high-status occupational classes for Black workers, with improved access to low-status occupational classes for white workers; gender inequity still exists Scenario B: Improved access to high-status occupational classes for Black workers, with improved access to low-status occupational classes for white workers; gender inequity no longer exists Scenario C: Black men access occupational classes similarly to white men in the status quo scenario, while Black women access occupational as white women Scenario D: All workers access occupational classes like white men in the status quo scenario

**Figure ****3** shows the hypertension prevalence trends among the Black and white healthcare workforces under the stutus quo and four desegregation scenarios. The prevalence averaging from age 25 to 64 for each scenario is highlighted at the top of each panel. The average hypertension prevalence by age group for all scenarios is also displayed in **Table **3. For the status quo scenario, the average hypertension prevalence was 14.3% for the white and 16.3% for the Black workforces.

**Table 3:** Average prevalence (cases per 100) of hypertension among the Black and white healthcare workforces by age group under the status quo and four counterfactual scenarios

**Age Group**	**Status Quo**		**Scenario A**^a^		**Scenario B**^b^		**Scenario C**^c^		**Scenario D**^d^	
	**White**	**Black**	**White**	**Black**	**White**	**Black**	**White**	**Black**	**White**	**Black**
25–29	4.0	5.0	4.0	4.7	4.0	4.7	4.0	4.7	3.8	4.5
30–34	6.2	7.6	6.3	7.2	6.3	7.2	6.2	7.1	5.9	6.7
35–39	9.0	11.0	9.1	10.5	9.1	10.4	9.0	10.3	8.5	9.8
40–44	12.2	14.7	12.4	14.1	12.4	14.0	12.2	13.9	11.7	13.3
45–49	15.7	18.3	15.9	17.7	15.9	17.6	15.7	17.5	15.0	16.8
50–54	19.2	21.7	19.4	21.1	19.3	21.0	19.2	20.9	18.5	20.2
55–59	22.5	24.7	22.7	24.2	22.6	24.1	22.5	24.0	21.8	23.4
60–64	25.5	27.5	25.7	26.9	25.7	26.9	25.5	26.8	24.9	26.2
25–64	14.3	16.3	14.4	15.8	14.4	15.8	14.3	15.6	13.8	15.1

a. Improved access to high-status occupational classes for Black workers, with improved access to low-status occupational classes for white workers; gender inequity still exists

b. Improved access to high-status occupational classes for Black workers, with improved access to low-status occupational classes for white workers; gender inequity no longer exists

c. Black men access occupational classes similarly to white men in the status quo scenario, while Black women access occupational as white women

d. All workers access occupational classes like white men in the status quo scenario.

The second and third panel of **Fig. ****3** shows the hypertension prevalence trends among the Black and white healthcare workforces for scenarios A and B simulations. When the Black and the white workers were allocated to occupational classes using the population-average probabilities, we observed the narrowing of the Black–white inequity gap in the hypertension prevalence. In scenario A, where gender inequity still exists, the average hypertension prevalence was 14.4% and 15.8% for the white and the Black workforces, respectively. We also observed a similar prevalence gap in scenario B, where race and gender inequities were absent from the healthcare workforces. In both scenarios, the decrease in racial inequity results from the Black workers becoming healthier and the white workers facing a greater risk of hypertension.

The fourth panel of **Fig. ****3** shows the hypertension prevalence trends in the scenario where the probabilities of entering a given occupational class for the Black workers were set equal to those of the white workers in the status quo scenario (scenario C). Consistent with results from scenarios A and B, we observed the narrowing of the Black–white inequity in hypertension. However, the reduction we observed in this scenario resulted from the decreased hypertension prevalence for the Black workforce (average prevalence of 15.6%) without changing the hypertension prevalence in the white workforce (average prevalence of 14.3%). In scenario D, where all the workers, regardless of their race and gender, have the same occupational class access as the white men in the status quo scenario, the average hypertension prevalence among the Black (15.1%) and the white workforces (13.8%) decreases (the fifth panel of **Fig. ****3**). The decrease in hypertension prevalence among the white workforce in this scenario is attributable to a reduction among the white female workers as they have greater access to higher-status, lower-risk occupational classes than in the segregated workforce.

Results from our sensitivity analyses show consistent trends with our base-case simulations. Even with ± 25% uncertainty around the probabilities of being prescribed hypertension medication by race and the transition probabilities from the hypertensive to controlled states by adherence levels, the decrease of the racial gap in the hypertension prevalence between the Black and white workforces are similarly evident in both of the sensitivity analyses (**Table **4).

**Table 4:** Sensitivity analyses

Scenario	Workforce	Base Case	± 25% Probability of being prescribed hypertension medication	± 25% Transition probability: Hypertensive → Controlled
Status Quo	White	14.3	(12.9, 16.3)	(12.7, 16.6)
	Black	16.3	(14.8, 18.5)	(14.3, 19.2)
Scenario A^a^	White	14.4	(13.1, 16.5)	(12.8, 16.8)
	Black	15.8	(14.3, 18.0)	(13.9, 18.6)
Scenario B^b^	White	14.4	(13.0, 16.5)	(12.8, 16.8)
	Black	15.8	(14.3, 17.9)	(13.9, 18.6)
Scenario C^c^	White	14.3	(12.9, 16.3)	(12.7, 16.6)
	Black	15.6	(14.2, 17.8)	(13.7, 18.4)
Scenario D^d^	White	13.8	(12.4, 15.8)	(12.2, 16.0)
	Black	15.1	(13.7, 17.2)	(13.3, 17.8)

a. Improved access to high-status occupational classes for Black workers, with improved access to low-status occupational classes for white workers; gender inequity still exists

b.Improved access to high-status occupational classes for Black workers, with improved access to low-status occupational classes for white workers; gender inequity no longer exists

c.Black men access occupational classes similarly to white men in the status quo scenario, while Black women access occupational as white women

d. All workers access occupational classes like white men in the status quo scenario.

## Discussion

The role of work as a multi-faceted driver of health inequities has received more attention from scholars in light of the COVID-19 pandemic and the significant health risks faced by healthcare workers (Kambhampati et al., 2020). Nevertheless, such inequities have existed for years, and the lack of resources to protect workers from diseases is only one contributing factor among many. Knowledge of how occupational segregation transpires into racialized occupational health risk exposure and health inequities among workers is limited in the current literature. To address this gap, we utilized a microsimulation to integrate information about 1) occupational segregation by race and gender, 2) patterns of risk exposure by occupational classes, and 3) biological and social production of workers’ health. We examined the extent to which occupational segregation contributes to the persistent racial inequity in hypertension, one of the most prevalent chronic conditions among US healthcare workers. Our microsimulation experiment, which allows only occupational class access for Black and white healthcare workers at the start of their career to varying across scenarios, reveals several interesting findings.

First, we observed an approximate one percentage point decrease in the average hypertension prevalence among the Black workforce when occupational segregation is absent. This finding supports previous evidence on the adverse effects of occupational segregation on Black workers' health (Chung-Bridges et al., 2008; Meyer, 2014). Because our microsimulation created a laboratory-like environment where “everything else” that confounds the relationship between occupational segregation and hypertension inequity is minimized, our results are less vulnerable to unobserved biases than previous studies. Given the estimated 1.7 million Black healthcare workers in the US and the $1,920 excess health care expenses incurred by hypertensive patients annually (Kirkland et al., 2018), one percentage point decrease in the hypertension prevalence in the desegregated workforce could have translated into at least approximately $32 million of saving to society each year.

Second, we observed a decrease in hypertension among the Black workforce but an increase in hypertension among the white workforce when both groups were allocated to occupational classes using the race-independent and race-and-gender-independent population average probabilities relative to the status quo condition. We would likely observe these trends when the structural advantages for white workers created by white supremacy are eliminated completely. White supremacy is a sociopolitical system that socially, economically, and ideologically benefits European descendants and oppresses people in other racial groups (Malat et al., 2018). From white supremacy, the concept of whiteness was formed. Whiteness refers to the normalization of white racial identity throughout America's history, creating a culture where non-white persons are seen as inferior or abnormal (Bonilla-Silva, 2004). In *Black Reconstruction in America, 1860–1880*, W.E.B. Du Bois coined the term “wage of whiteness” to characterize psychological benefits that the white working-class received because of their racial status in a racialized society (Du Bois, 1998). Du Bois stated, “It must be remembered that the white group of laborers, while they received a low wage, were compensated in part by a sort of public and psychological wage,” explaining why the white working-class united with the capitalist whites to lay the structural foundation of modern white supremacist policies and practices, even though they were exploited by capitalism and had as similar economic experience as Black workers. Consistent with Du Bois’ view, we characterize the margin of health gain that white workers earn on Black workers' back because of occupational segregation in the healthcare organizations as the physical “wage of whiteness.” The eradication of white supremacy and the dismantling of labor market mechanisms that uphold occupational segregation are widely supported philosophically by a large majority of healthcare organizations (Alexis et al., 2020). Yet, the actual implementation of interventions designed to desegregate the healthcare workforce often progresses at a slow pace (Association for American Medical Colleges, 2020). The physical “wage of whiteness” enjoyed by workers at the top of the organizational hierarchy, especially those who believe this advantage will be taken away from them in the desegregated workplace, may be one of the critical resisting forces underlying the persistent occupational segregation in the healthcare workforces.

Third, we observed different levels of decrease in the hypertension inequity between the Black and white healthcare workforces across the four desegregation scenarios. Generally, we observed a greater decrease in hypertension when barriers to occupational class access are eliminated jointly for Black and female workers than when only racial barriers are eliminated. These findings reiterate the intersectionality of race and gender in the labor market process and their joint role in the social production of the workers’ health. Our study also reveals different degrees to which population health is improved depending on how desegregation is conducted. Taking the seats that should have belonged to Black and female workers back from white and male workers (scenario A and B) leads to narrowing the racial gap in hypertension, but with white and male workers facing a greater risk of hypertension. On the other hand, by making more seats available and prioritizing those seats for Black and female workers (scenario C and D), the racial gaps in hypertension will decrease due to decreasing hypertension risks for both Black and white workforces. Workforce desegregation policies are known commonly as “diversity, equity, and inclusion (DEI) policies” and can target different stages of the labor market process. For example, a series of laws classified as affirmative action has been implemented to improve diversity in higher education (i.e., the key requirement for high-status healthcare occupations) and to ensure that racial distribution of workers employed by government contractors reflects the diverse community served by these organizations (Arcidiacono et al., 2015; Holzer, Harry; Neumark, 1999; Kurtulus, 2016). The American Association of Medical Colleges and other health professional organizations also established pathway and mentoring programs to increase the physician workforce from historically underrepresented backgrounds (Fontenot & McMurray, 2020; Jacob, 2015; Merchant & Omary, 2010). Because these DEI policies are carried out in concert with continuing efforts to eliminate the shortage of high-status healthcare workers (e.g., a partnership between healthcare and institutions to increase enrollment, federal legislatures to increase residency positions eligible for graduate medical education under Medicare, accelerated baccalaureate and master degree in nursing programs, the National Health Services Corp Student to Service Loan Repayment Program, etc.), DEI policies should not create adverse effects on the health of the overall healthcare workforce. Future studies should examine the health impact of specific DEI policies, as some DEI policies are more effective in desegregating the workforce than others (Kalev et al., 2006). Because desegregation of the healthcare workforce may result in restructuring healthcare organizations, future studies should also examine how various desegregation approaches affect patient care delivery and quality.

The results presented in this paper are an early iteration of our effort to improve knowledge of the role of work as a social determinant of health and health inequities. Our current study has a few limitations. First, the model we used to mimic occupational segregation in the healthcare workforce and the pathogenesis of hypertension among healthcare workers simplified much more complex real-world processes. Other models that operationalize these processes differently or use different model assumptions may lead to a different conclusion. We encourage other researchers to modify our model structure or assess the impact of desegregation on hypertension inequity under the different model assumptions to validate our findings. Second, our prediction of hypertension onset among workers considered the effect of job insecurity, job loss, and psychosocial work environment—the heavily researched occupational risk factors that are prevalent in the low-status occupational classes. Our current model did not focus on the potential effect of work-related stressors reported in other previous studies to be prevalent among high-status workers, especially among non-white workers. Examples of such stressors include hiring and workplace discrimination, the experience of tokenism, John Henryism, and increased work-home life interference (Bonham et al., 2004; Braboy Jackson et al., 1995; Grace & VanHeuvelen, 2019; Quillian et al., 2017). We also did not explicitly model the effect of workers’ education, income level, and seniority in the workplace on their hypertension status. Hence, our results may not be entirely free of unmeasured biases. Our future efforts are to update our current model to better mimic the intricate social production of workers’ health processes, incorporate the interplay between various stressors and socioeconomic correlates of health, and examine the health impact of desegregation with greater precision.

## Conclusion

We highlight the role of occupational segregation as a driver of health inequities in Black and white workforces and provide the first evidence that workforce desegregation will reduce workers’ health inequities and improve the overall population health. We found that desegregating the healthcare workforce, of which the primary intent is to have a workforce that reflects the community that the healthcare workers serve, can improve the workers’ health. However, more evidence of various desegregation effectiveness in the real work is needed. The policy window to dismantle structural racism and sexism in the labor markets—the key force underlying occupational segregation—is now. Policymakers can adapt the innovative microsimulation approach used in our study to predict the potential impact of labor market and health equity policies.

## Supplementary Information

Below is the link to the electronic supplementary material.

Supplementary file1 (PDF 398 KB)

## Data Availability

Access is granted on a case-by-case basis. Please send a request to the corresponding author.

## Code Availability

Access is granted on a case-by-case basis. Please send a request to the corresponding author.
