# Supplementary material for: Occupational Segregation And Hypertension Inequity: The Implication Of The Inverse Hazard Law Among Healthcare Workers
Source: J Econ Race Policy. 2022 Mar 22;5(4):267–82. doi: 10.1007/s41996-022-00098-5 (PMC8938730; doi:10.1007/s41996-022-00098-5)
Supplement: Supplementary file 1 — Supplementary file1 (PDF 398 KB) [file 41996_2022_98_MOESM1_ESM.pdf]

ONLINE RESOURCE

Online Resource 1: Healthcare occupational classes and their proportional representation in the white and Black healthcare workforces based on the 2017 American Community Survey.

Online Resource 2: Prevalence of hypertension among healthcare workers by occupational class and race

Online Resource 3: Predicting transition probabilities to the prehypertensive and hypertensive states

Online Resource 4: Tracking hypertension risk factors and hypertension status overtime

Online Resource 5: Descriptive statistics of job demand, job control, and support measures by occupational class.

Online Resource 6: Status-quo simulation

Online Resource 1: Healthcare occupational classes and their proportional representation in the white and Black healthcare workforces based on the 2017 American Community Survey.

**Class**	**2010 Standard Occupational Code**	**Occupation**	**white (N=6,864,442)**	**Black (N=1,719,595)**
Health Diagnosing (Doctoral Degree)	29-1011	Chiropractors	12.5%	3.7%
	29-1020	Dentists		
	29-1041	Optometrists		
	29-1051	Pharmacists		
	29-1060	Physicians and Surgeons		
	29-1081	Podiatrists		
	29-1181	Audiologists		
	29-1131	Veterinarians		
Health Treating (Master's Degree)	29-1122	Occupational Therapists	43.0%	22.6%
	29-1123	Physical Therapists		
	29-1124	Radiation Therapists		
	29-1125	Recreational Therapists		
	29-1126	Respiratory Therapists		
	29-1031	Dieticians and Nutritionists		
	29-1071	Physician Assistants		
	29-1141	Registered Nurses*		
	29-1127	Speech Language Pathologists		
	29-112X; 29-1129	Other Therapists, Including Exercise Physiologists		
	29-1151; 29-1141	Nurse Anesthetists		
	29-11XX; 29-1141	Nurse Practitioners and Nurse Midwives		

	29-1199	Health Diagnosing and Treating Practitioners, All Other		
Health Technicians (Bachelor's Degree)	29-2010	Clinical Laboratory Technologists and Technicians	22.5%	24.2%
	29-2021	Dental Hygienists		
	29-2030	Diagnostic Related Technologists and Technicians		
	29-2041	Emergency Medical Technicians and Paramedics		
	29-2050; 29-2050	Health Practitioner Support Technologists and Technicians		
	29-2061	Licensed Practical and Licensed Vocational Nurses		
	29-2071	Medical Records and Health Information Technicians		
	29-2081	Opticians, Dispensing		
	29-2090	Miscellaneous Health Technologists and Technicians		
	29-9000	Other Healthcare Practitioners and Technical Occupations		
Healthcare Aides (Less than Bachelor's Degree)	31-1010	Nursing, Psychiatric, and Home Health Aides	21.9%	49.5%
	31-2010	Occupational Therapy Assistants and Aides		
	31-2020	Physical Therapist Assistants and Aides		
	31-9011	Massage Therapists		
	31-9091	Dental Assistants		
	31-9092; 31-909X	Medical Assistants		

	31-9094; 31-909X	Medical Transcriptionists		
	31-9095; 31-909X	Pharmacy Aides		
	31-9096; 31-909X	Veterinary Assistants and Laboratory Animal Caretakers		
	31-9097; 31-909X	Phlebotomists		
	31-909X; 31-909X	Healthcare Support Workers, All Other, Including Medical Equipment Preparers		

Footnote

We grouped healthcare workers based on the educational requirement for their professional tasks and licensing. We put registered nurses (RN) in the "health treating" class, which requires a master's degree. In reality, RN is a broadly defined occupation; RN can hold an educational degree ranging from an associate to a doctoral level. Our decision to designate RN as a master-level "health treating" occupation was motivated by our need to differentiate RN, licensed practical nurses (LPN), and nursing aides into three separate occupational classes, reflecting different levels of work authority, occupational prestige, and average wage. This classification is consistent with the one used previously by Chou and Johnson (2008). However, their classification focuses on job function rather than workers' position along the professional hierarchy (Chou & Johnson, 2008).

Online Resource 2: Prevalence of hypertension among healthcare workers by occupational class and race

We calculated the prevalence of hypertension among the US healthcare workforce using the 2014 to 2018 National Health Interview Survey data (NHIS). Sample individuals were asked, "During the past 12 months, have you had hypertension, also called high blood pressure"? The NHIS assigned sample individuals to the 1995 Occupational Code (OCC). For our analysis, we compare the 5-year prevalence of hypertension by race for individuals with the OCC 222 ("Health Diagnosing Occupations"), 223 ("Health Assessing and Treating Occupations), 301 (Health Technologist/Technician Occupations"), and 805 ("Health Service Occupations"). Note that the occupational classes based on the OCC are slightly different from those in Online Supplement 1 and may explain an inconsistent hypertension gradient by occupational class.

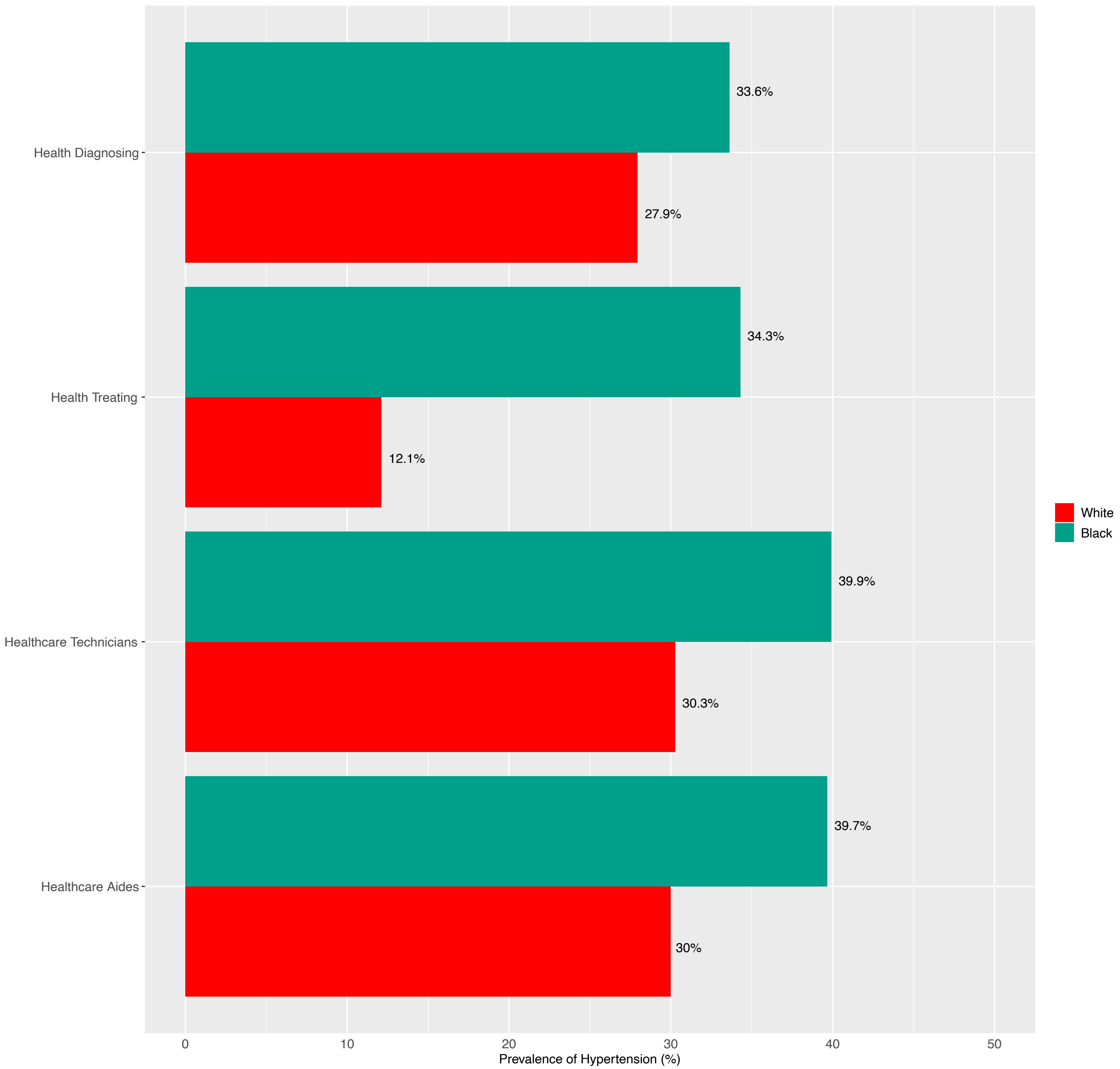
Health Diagnosing
Health Treating
Healthcare Technicians
Healthcare Aides
33.6%
27.9%
34.3%
12.1%
39.9%
30.3%
39.7%
30%
0
10
20
30
40
50
Prevalence of Hypertension (%)
White
Black

Online Resource 3: Predicting transition probabilities to the prehypertensive and hypertensive states

At the start of our project, hypertension risk equations that incorporate work-related characteristics were not available. To our knowledge, this is still the case now. Thus, we estimated two equations to predict the probabilities of prehypertension and hypertension onset among workers using the data from the Coronary Artery Risk Development in Young Adults (CARDIA) study. Details about the study design and data collection protocols for the CARDIA study are available elsewhere (Friedman et al., 1988). To derive these equations, we linked CARDIA participants' health and work characteristics collected as a part of the Year 15 follow-up assessment with the job title-based measures of the psychosocial work environment (PWE) that we derived from the job rating data from the Occupational Information Network (O*Net) database version 3.1 (O*NET Resource Center, 2019). We measured three dimensions of the PWE. The job demand measure includes ratings of workers 'ability to shift back and forth between activities/source of information, ability to concentrate on a task over a period of time without being distracted, the seriousness of the error, and the importance of being accurate in this job (score: 0-4; Cronbach's alpha=0.69) (Cifuentes et al., 2007). The job control measure includes ratings of whether the job makes use of workers' abilities, ability to try out their ideas, ability to make decisions on their own, and ability to plan their work with little supervision (score: 0-4; Cronbach's alpha=0.96) (Cifuentes et al., 2007). The support measure uses rating scales of whether workers on this job have supervisors who train their workers well and back them up with management (score: 0-2; Cronbach's alpha=0.78) (McCluney, Schmitz, Hicken, & Sonnega, 2018). These PWE measures were used in conjunction with participants' employment

status. We assigned these PWE scores only to participants who worked full-time and part-time in Year 15. We assigned zero for all three measures to the unemployed participants.

We fitted the logistic regressions to estimate the probability of prehypertension and hypertension five years later (Year 20 follow-up assessment). We used a backward selection algorithm and selected the model with the lowest Bayesian Information Criteria (BIC) as the most parsimonious one. The Harrell c-statistic and the Hosmer-Lemeshow goodness-of-fit statistic were used to assess model discrimination and calibration, respectively. Below are the equations we used to predict the five-year probabilities of prehypertension (c-statistic=0.7; Hosmer-Lemeshow $\chi 2$=11.8) and hypertension (c-statistic=0.8; Hosmer-Lemeshow $\chi 2$=9.2) in our simulation.

$$Logit(5-year\ hypertension) = -12.07 + 2.497 LogBMI + 0.775 Current\ Smoker + 1.710 Currently\ Prehypertensive + 0.656 Family\ History\ of\ Hypertension - 0.059 Work\ Full\ Time - 0.135 Work\ Part\ Time + 0.167 Job\ Demand - 0.347 Job\ Control - 0.160 Support + 0.024 Age + 0.197 Women \quad (1)$$

$$Logit(5-year\ prehypertension) = -5.106 + 0.013 LogBMI - 0.424 Current\ Smoker + 1.155 Currently\ Prehypertensive + 0.509 Family\ History\ of\ Hypertension + 0.361 Work\ Full\ Time + 1.470 Work\ Part\ Time - 0.278 Job\ Demand - 0.657 Job\ Control - 0.206 Support - 0.078 Age + 0.004 Women \quad (2)$$

Online Resource 4: Tracking hypertension risk factors and hypertension status overtime

Our model tracked changes in exposure to hypertension risk factors in equations (1) and (2) every cycle to determine hypertension status for the simulated workers. As the simulation progresses, the workers become one year older at the end of each cycle. Besides age, smoking status, physical activity, BMI, and employment status may change from one cycle to the next throughout the simulation period.

Smoking Status and Physical Activity

Workers may transition between the non-smoker, smoker, and quit state (Figure 4) and between the physically inactive and active state (Figure S4.2). We modeled these transitions with the state-transition model (STM). Both STMs used a one-year cycle. Transition probabilities for the smoking and physical activity STMs and their sources are shown in Table 1.

Fig 4: State transition diagram for smoking status

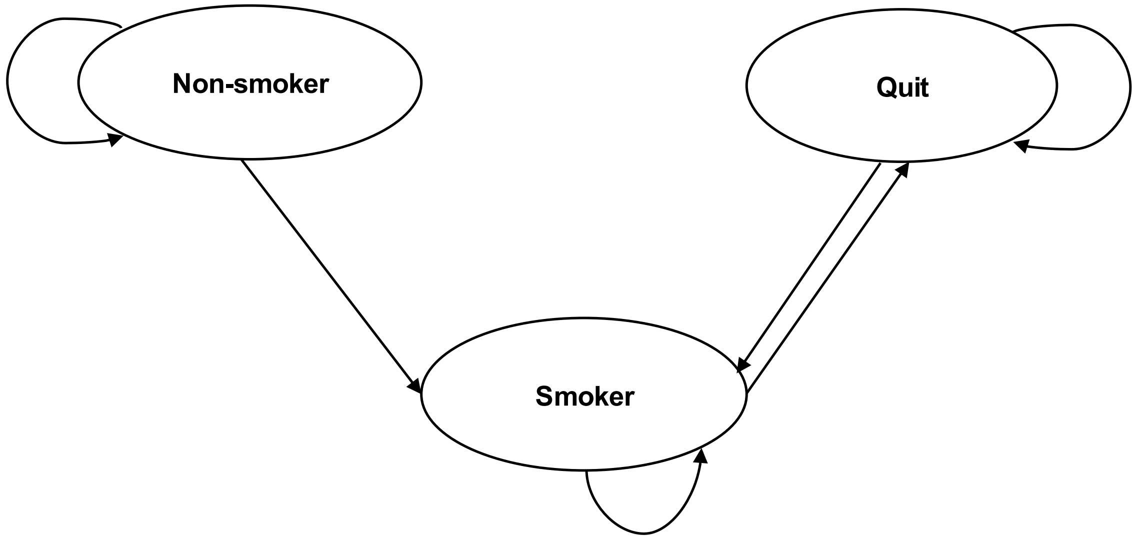

Fig 5: State transition diagram for physical activity

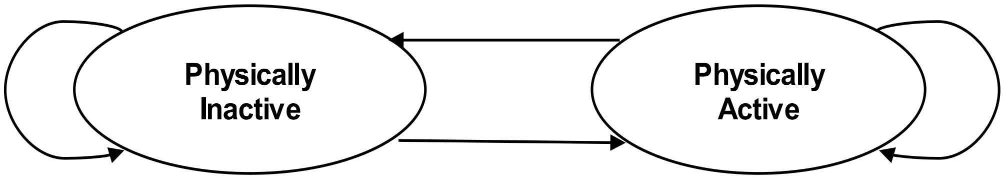

Body Mass Index and Employment Status

To track changes in BMI and employment status over time, we estimated autoregressive regression models to predict current BMI level and employment status conditioning on BMI level from two years ago and employment status one year ago, respectively.

The autoregressive linear regression model of the logarithm of BMI was estimated using the CARDIA data and is shown below.

$$LogBMI = 0.173 - 0.001Age - 0.036Physically\ Active - 0.007Current\ Smoker + 0.002Former\ Smoker + 0.978LogBMI\ Two\ Years\ Ago \quad (3)$$

The autoregressive multinomial regression models predicting whether healthcare workers in specific occupational class work full-time, part-time, or are unemployed were estimated using the American Community Survey (ACS) data (2012-2017).

Health Diagnosing Workers

$$\ln\left(Pr\ (full\ time)/Pr\ (unemployed)\ \right) = -15.561 - 0.024Age - 0.860Women - 0.466Black + 22.332Employed\ last\ year \quad (4)$$

$$\ln\left(Pr\ (part\ time)/Pr\ (unemployed)\ \right) = -51.658 + 0.009Age + 0.385Women - 1.065Black + 54.453Employed\ last\ year \quad (5)$$

Health Treating Workers

$$\ln\left(Pr\ (full\ time)/Pr\ (unemployed)\ \right) = -14.225 - 0.016Age - 0.110Women - 0.140Black + 19.519Employed\ last\ year \quad (6)$$

$$\ln\left(Pr\ (part\ time)/Pr\ (unemployed)\ \right) = -14.704 - 0.007Age + 1.015Women - 0.872Black + 17.333Employed\ last\ year \quad (7)$$

Healthcare Technicians

$$\ln\left(Pr\ (full\ time)/Pr\ (unemployed)\ \right) = -13.624 - 0.001Age - 0.071Women - 0.203Black + 17.474Employed\ last\ year \quad (8)$$

$$\ln\left(Pr\ (part\ time)/Pr\ (unemployed)\ \right) = -14.794 + 0.001Age + 1.042Women - 0.649Black + 16.357Employed\ last\ year \quad (9)$$

Healthcare Aides

$$\ln\left(Pr\ (full\ time)/Pr\ (unemployed)\ \right) = -13.624 - 0.001Age - 0.071Women - 0.203Black + 17.474Employed\ last\ year \quad (10)$$

$$\ln\left(Pr\ (part\ time)/Pr\ (unemployed)\ \right) = -14.794 + 0.001Age + 1.042Women - 0.649Black + 16.357Employed\ last\ year \quad (11)$$

Other Characteristics

Workers' race, gender, occupational class, and family history of hypertension do not change over time. Therefore, the baseline characteristics were used to estimate the relevant probabilities throughout the simulation in all cycles. As mentioned above, the PWE scores are conditioned on the employment status. For example, suppose unemployed workers become

employed either full-time or part-time. In that case, the model will “turn on” the PWE scores and use them to predict the probabilities of prehypertension and hypertension onset for that cycle.

Online Resource 5: Descriptive statistics of job demand, job control, and support measure by occupational class.

We created the empirical distribution of job demand, job control, and support for all classes of healthcare workers. We linked the SOC-based PWE scores described earlier to the representative sample of healthcare workers sampled to participate in the ACS (2012-2017). Below are the descriptive statistics of the PWE scores from our empirical distributions. In our simulation model, we randomly drew the PWE scores for each simulated worker from the distributions specific to their occupational class.

Occupational Class	PWE Dimension	Mean	SD	Min	Q1	Q2	Q3	Max
Health Diagnosing	Job Demand	2.25	0.17	1.54	2.22	2.32	2.32	2.57
	Job Control	3.47	0.23	3.00	3.55	3.57	3.57	3.72
	Support	0.78	0.10	0.60	0.79	0.79	0.79	1.02
Health Treating	Job Demand	2.13	0.25	1.46	2.18	2.18	2.18	3.13
	Job Control	2.71	0.21	2.20	2.62	2.62	2.62	3.30
	Support	1.03	0.02	1.00	1.02	1.02	1.02	1.17
Healthcare Technicians	Job Demand	2.12	0.16	1.82	2.08	2.12	2.12	2.40
	Job Control	2.28	0.28	1.99	2.15	2.15	2.39	3.02
	Support	1.14	0.09	0.90	1.07	1.12	1.20	1.26
Healthcare Aides	Job Demand	1.85	0.28	1.47	1.67	1.67	2.07	2.45
	Job Control	1.63	0.09	1.57	1.58	1.58	1.70	1.97
	Support	1.22	0.05	1.15	1.19	1.19	1.25	1.35

Online Resource 6: Status-quo simulation

Figure 6 shows the trends that emerged from our model under the status-quo condition. The observed outcomes for the Black and the white healthcare workforces are colored in cyan and red, respectively. Throughout most of the follow-up period, the number of hypertensive cases was consistently higher among the Black than the white healthcare workforces. As both workforces approached their retirement age, the number of hypertensive cases started to equalize (upper left panel). However, looking at the number of hypertension cases alone can be misleading. Because the death rate for the Black workers was higher than for the white workers, there were almost always fewer Black workers alive who could transition into the hypertensive state during the follow-up period than the white workers (upper right panel). The lower panel shows the prevalence of hypertension, which was calculated as the number of hypertensive workers divided by the number of alive workers, provided a better representation of the racial inequity in hypertension among the two workforces. Specifically, the prevalence of hypertension averaging from age 25 to 64 for the status-quo (occupational segregation) was 14.3% for the white and 16.3% for the Black workforces

Fig 6: Status-quo simulation outputs

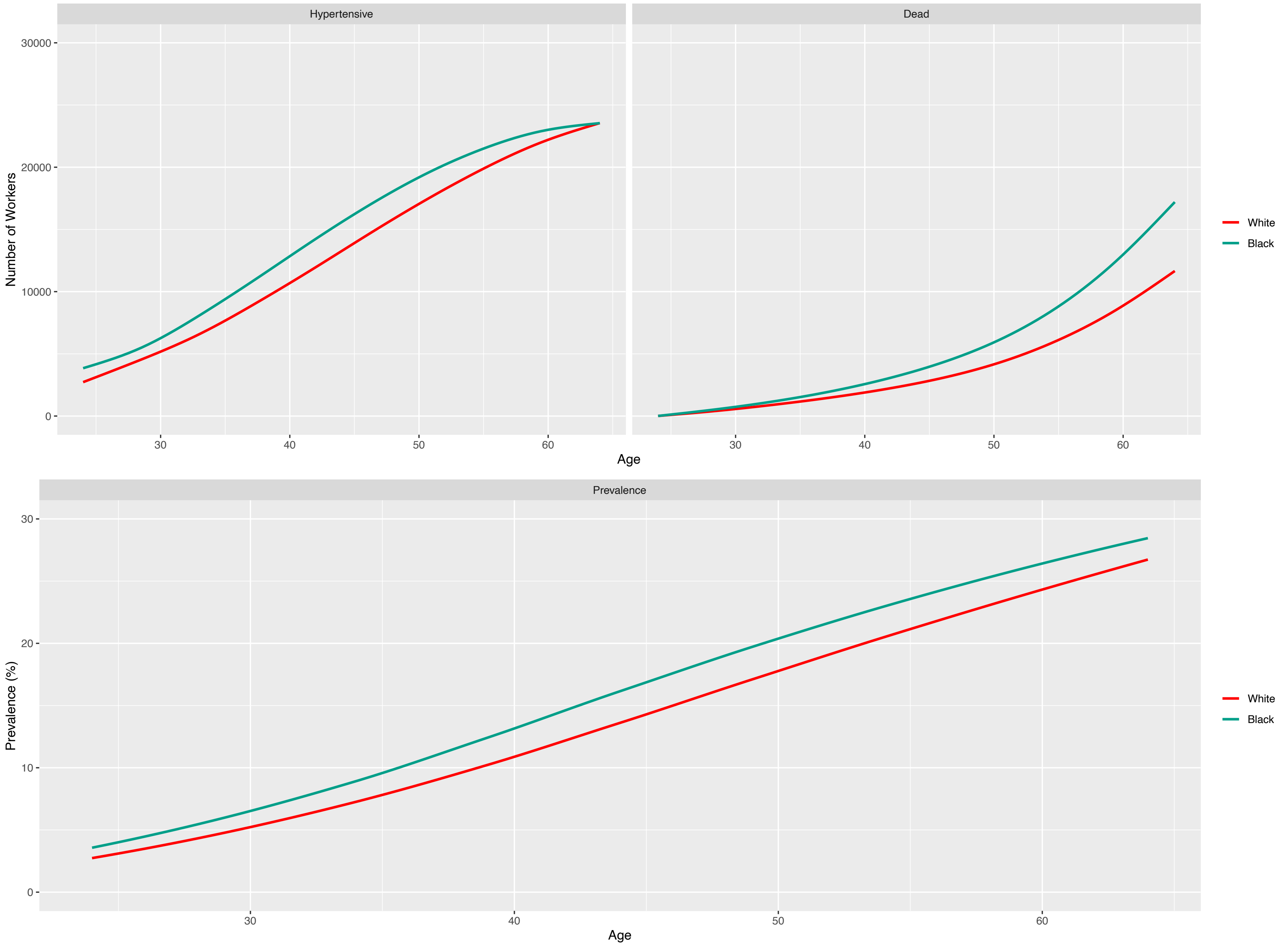

ADDITIONAL REFERENCES

Chou, C.-F., & Johnson, P. J. (2008). Health Disparities among America's Health Care Providers: Evidence from the Integrated Health Interview Series, 1982 to 2004. *Journal of Occupational and Environmental Medicine*, *50*(6), 696–704. https://doi.org/10.1097/JOM.0b013e31816515b5

Cifuentes, M., Boyer, J., Gore, R., D'Errico, A., Tessler, J., Scollin, P., … Team, P. in H. R. (2007). Inter-method Agreement between O*NET and Survey Measures of Psychosocial Exposure among Healthcare Industry Employees. *American Journal of Industrial Medicine*, *50*(7), 545–553. https://doi.org/10.1002/ajim.20480

Egan, B. M., & Stevens-Fabry, S. (2015). Prehypertension — Prevalence, Health Risks, and Management Strategies. *Nature Reviews Cardiology*, *12*(5), 289–300. https://doi.org/10.1038/nrcardio.2015.17

Friedman, G. D., Cutter, G. R., Donahue, R. P., Hughes, G. H., Hulley, S. B., Jacobs David R, J., … Savage, P. J. (1988). CARDIA: Study Design, Recruitment, and Some Characteristics of the Examined Subjects. *Journal of Clinical Epidemiology*, *41*(11), 1105–1116.

McCluney, C. L., Schmitz, L. L., Hicken, M. T., & Sonnega, A. (2018). Structural Racism in the Workplace: Does Perception Matter for Health Inequalities? *Social Science and Medicine*, *199*, 106–114. https://doi.org/10.1016/j.socscimed.2017.05.039

O*NET Resource Center. (2019). O*NET Resource Center. Retrieved July 9, 2019, from https://www.onetcenter.org/

Samanic, C. M., Barbour, K. E., Liu, Y., Fang, J., Lu, H., Schieb, L., & Greenlund, K. J. (2020). Prevalence of Self-Reported Hypertension and Antihypertensive Medication Use Among Adults-United States, 2017. *Morbidity and Mortality Weekly Report*, *69*(14), 393–398.
